# An Open Source Business Model for Malaria

**DOI:** 10.1371/journal.pone.0117150

**Published:** 2015-02-06

**Authors:** Christine Årdal, John-Arne Røttingen

**Affiliations:** 1 Department of International Public Health, Norwegian Institute of Public Health, Oslo, Norway; 2 Section for Global Health, Norwegian Knowledge Centre for the Health Services, Oslo, Norway; 3 Division of Infectious Disease Control, Norwegian Institute of Public Health, Oslo, Norway; 4 Department of Health Management and Health Economics, Institute for Health and Society, University of Oslo, Oslo, Norway; 5 Department of Global Health and Population, Harvard School of Public Health, Boston, Massachusetts, United States of America; University of Sydney, AUSTRALIA

## Abstract

Greater investment is required in developing new drugs and vaccines against malaria in order to eradicate malaria. These precious funds must be carefully managed to achieve the greatest impact. We evaluate existing efforts to discover and develop new drugs and vaccines for malaria to determine how best malaria R&D can benefit from an enhanced open source approach and how such a business model may operate. We assess research articles, patents, clinical trials and conducted a smaller survey among malaria researchers. Our results demonstrate that the public and philanthropic sectors are financing and performing the majority of malaria drug/vaccine discovery and development, but are then restricting access through patents, ‘closed’ publications and hidden away physical specimens. This makes little sense since it is also the public and philanthropic sector that purchases the drugs and vaccines. We recommend that a more “open source” approach is taken by making the entire value chain more efficient through greater transparency which may lead to more extensive collaborations. This can, for example, be achieved by empowering an existing organization like the Medicines for Malaria Venture (MMV) to act as a clearing house for malaria-related data. The malaria researchers that we surveyed indicated that they would utilize such registry data to increase collaboration. Finally, we question the utility of publicly or philanthropically funded patents for malaria medicines, where little to no profits are available. Malaria R&D benefits from a publicly and philanthropically funded architecture, which starts with academic research institutions, product development partnerships, commercialization assistance through UNITAID and finally procurement through mechanisms like The Global Fund to Fight AIDS, Tuberculosis and Malaria and the U.S.’ President’s Malaria Initiative. We believe that a fresh look should be taken at the cost/benefit of patents particularly related to new malaria medicines and consider alternative incentives, like WHO prequalification.

## Introduction

Malaria is an ancient, global scourge that continues to plague the world today. Up until the mid-19^th^ century it was endemic in most countries, placing 90% of the world’s population at risk [1]. The Centers for Disease Control and Prevention was established in 1946 to combat malaria in the U.S. [2]. By the 1960s North America and Europe had been declared malaria free by the World Health Organization (WHO) [3].

Today malaria remains a leading cause of death globally (ranked 11^th^ in 2010) [4], killing 627,000 of the estimated 207 million infected [5]. It is a leading killer of children under the age of five [4]. However, tremendous progress has been made. Between 1955 and 2012 more than 50 countries have eliminated malaria [6]. South Africa has reduced malaria mortality by 85% in the last 12 years and anticipates eliminating it by 2018 [7].

Investments in both malaria control and R&D have dramatically increased in the last decade with the establishment of organizations like The Global Fund to Fight AIDS, Tuberculosis and Malaria, UNITAID, Roll Back Malaria and Medicines for Malaria Initiative (MMV). New medicines have become available, most notably artemisinin combination treatments (ACTs), and there continue to be significant advancements, such as recent progress in producing synthetic artemisinin [8]. GlaxoSmithKline (GSK) has sought regulatory approval for the first ever malaria vaccine, RTS,S, against the *Plasmodium falciparum* malaria parasite, which has demonstrated about 50% efficacy in young children [9].

The G-Finder report found that US$ 559 million was spent on malaria R&D in 2011 [10]. This decreased to US$ 543 million in 2012 [11]. Comparatively US$ 1,029 million was spent on HIV/AIDS specifically targeted at developing-country R&D needs [10]. The current level of investment in malaria R&D is insufficient if the aim is, as has been called for [12], to eradicate malaria. As the parasites adapt to current therapies with the emergence of resistance, new medicines are needed. Improved, rapid diagnostics are needed that use urine samples instead of blood as well as diagnostics with higher sensitivity [13]. New vector control products are needed to reduce the mosquito population, including better forms for indoor residual spraying. Ultimately more effective vaccines are needed.

The vast majority (82%) of investment in malaria R&D in 2011 came from the public or philanthropic sectors [10]. This is the case because there are market failures when it comes to antimalarials—where the profit potential of new treatments does not outweigh the costs necessary to develop them. MMV is the principal product development partnership active within malaria drug discovery and development. It is funded by government agencies, private foundations, international organizations, corporations, corporate foundations, and private individuals. None of its donors are pharmaceutical or vaccine manufacturers, but it partners with the pharmaceutical industry in its research portfolio [14]. The magnitude of these in-kind contributions is difficult to determine. The PATH Malaria Vaccine Initiative, funded by the Bill & Melinda Gates Foundation, is the main product development partnership active in malaria vaccine discovery and development.

Open source drug discovery, which is one approach to open knowledge innovation [15], is a model based upon the open source software development movement, where scientists contribute collaboratively across geographic areas to produce freely-available results towards the ultimate development of new drugs, vaccines or diagnostics. The non-profit research organization, Results for Development Institute, has defined an open source drug discovery project as one where: [16]

1. The data are open access, meaning that anyone can view the data free-of-charge.
2. There is a forum for open collaboration (across organizational and geographical boundaries).
3. A set of rules that mandate “openness” govern the project.

We evaluate existing efforts to discover and develop new drugs and vaccines for malaria to determine whether malaria R&D would benefit from an open source approach and how such a business model may operate. Therefore, we first assess how “open” malaria R&D is today through the following research questions:

- Are malaria research results (both data and biomedical samples) openly available?
- Is malaria research performed collaboratively and what are scientists’ and organizations’ motivations for participation?
- To what extent are intellectual property rights used to protect malaria R&D outputs?

Since drug discovery and development is an incremental effort, the answers to these questions will vary depending upon the phase of the pharmaceutical value chain. The value chain [17] (see Table 1) starts with basic research and ends with a finished product (either a new drug or vaccine).

**Table 1:** The Pharmaceutical Value Chain [17].

	Early Stage Discovery	Late Stage Discovery	Clinical Trials	Regulatory Approval
Description	Basic research (i.e., undirected research into the mechanisms that cause disease) and target identification and validation (i.e., identifying and validating points where drugs can intervene to disrupt disease)	Finding and optimizing lead compounds to ensure that they possess the properties needed to make an effective drug.	Determining the efficacy and safety of a drug/vaccine in people	Securing governmental authorization to sell a drug/vaccine

Finally, we apply the data we have gathered about the existing malaria R&D model and suggest how a more macro-level open source business model may benefit the overall process. Others have examined micro-level tactics to examine researcher engagement levels [18]. Our analyses and recommendations are not related to nor discuss particular open source malaria projects, such as “Open Source Malaria”, “OSDDm” or the Worldwide Antimalarial Resistance Network. We take a broader and more generic view, specifically through a macro-level business model. A business model describes how an organization “creates, delivers, and captures value” [19]. It is normally specific to one organization. However, we will use it more generally within the context of each phase of the pharmaceutical value chain, where multiple organizations may adopt the same model.

## Methods

We have classified organizations simply as either industry or non-industry. Industry is any for-profit company. Non-industry includes academic institutions, governments, non-profit research institutes, foundations, and product development partnerships (since their funding is largely public or philanthropic).

We gathered data by phase of the pharmaceutical value chain. For early stage drug/vaccine discovery, we extracted data for malaria from the G-Finder database across all its financial years (2007–2011) with the product type of “basic research”. (The 2012 G-Finder data had not yet been released when we gathered our data.)

We also extracted all articles from PubMed published in one year between April 29, 2012 and April 29, 2013 that contain the MeSH term “antimalarials/chemistry” (n = 161). This captures data for both early and late stage drug discovery, albeit only from those researchers that have published their work which may be a bias towards public sector researchers. From these articles we extracted:

- The corresponding author’s organization type (industry, non-industry or both)
- Number of different organizations represented by the co-authors (where departments at the same institution are counted separately)
- The organization type of all authors combined (industry, non-industry or both)
- The continents represented by the co-authors
- Type of funding received to perform the research (industry, non-industry or both)—where this information was not stated within the article, an e-mail was sent to the corresponding author requesting the funding source
- Whether the article was available open access

Non-duplicative corresponding authors (n = 143) were asked to complete an anonymous survey (see S1 Appendix) which included the researcher’s motivations for performing malaria research, his/her employer’s perceived motivation, use of patents, availability of biological samples or chemical/molecular compounds as well as perceptions to several concepts related to open source drug discovery. 38% (n = 54) responded to the survey after three reminders. We later refer to this group as “discovery researchers”.

Additionally for late stage drug and vaccine discovery and clinical trials, we extracted data for malaria from the G-Finder database across all financial years (2007–2011) with the product type of “drugs” and “vaccines”.

For clinical trials, we extracted all articles from PubMed published between April 29, 2012 and April 29, 2013 that contain the MeSH term “malaria” and were classified as a publication type “clinical trial” (n = 75). The same data as mentioned above was extracted from the article. Non-duplicative corresponding authors (n = 70) were asked to complete a similar anonymous survey to the one previously mentioned (see S1 Appendix). 20% (n = 14) responded to the survey after three reminders. We later refer to this group as “clinical trial researchers”.

We performed queries on July 3, 2013 for clinical trials registered WHO’s International Clinical Trials Registry Platform (ICTRP) (http://apps.who.int/trialsearch/). We searched for all trials for the condition of “malaria” that were registered from July 1, 2011 to July 3, 2013 (n = 196). We performed queries on July 2, 2013 for clinical trials registered in the U.S. National Institutes of Health trial database (clinicaltrials.gov). We searched for all trials for the condition of “malaria” that were received from July 1, 2011 to July 2, 2013 (n = 128). These should be a subset of the ICTRP trials; 103 of our ICTRP results had a source of clinicaltrials.gov.

For regulatory approval, we reviewed on June 17, 2013 all 31 manufacturer/drug combinations on the WHO List of Prequalified Medicinal Products (http://apps.who.int/prequal/) related to malaria. This is comprised of nine separate manufacturers for 21 different drug combination formulations.

We performed a patent search on July 5, 2013 in the US Patent and Trademark Office (http://patft.uspto.gov/). We searched for patents which contained the word "malaria" in their abstract and were applied for between 1993 and 2013 (n = 149). We also searched patent applications which contained the word "malaria" in their abstract and were applied for between 2011 and 2013 (n = 46). (There was no way that we could see to search for only applications in process since the Publication Document Kind Code does not appear to be updated with the current status). Four applications had been granted so these were removed from our data to avoid duplication.

To perform the market sizing for malaria medicines and vaccines, the suspected number of cases in 2011 per country was extracted from WHO’s *World Malaria Report 2012*, *Annex 6D—Reported malaria cases by species* [5]. However, since the annex did not report the suspected number of cases by malaria species, the country profiles from the report were used to extract the percentage of plasmodium species which were then applied to the total suspected cases.

The business model has been described according to Chesbrough’s and Rosenbloom’s standard functions [20]. These are:

- Value proposition: the value created through the service or product
- Market segment: the users of the service or product, why they use it and how the service or product generates revenue or value for them
- Value chain: the structure required to create and distribute the service or product (see Table 1)
- Cost structure and profit potential of the service or product
- Value network: the position of the innovator related to suppliers and customers
- Competitive strategy by which innovations are protected

We sought approval for our research portfolio (including the surveys) from the Norwegian Committees for Medical and Health Research. The Committee decided that our research did not require their ethical approval since we are studying collaboration amongst scientists and not patients. With that said, the survey responses were anonymous (controlled through the computer software, Questback) and it was stated in the invitation to participate that participation was completely voluntary.

## Results

We present our results by phase of the pharmaceutical value chain, i.e. early stage drug and vaccine discovery, late stage drug and vaccine discovery and clinical trials. Our results are summarized in Table 2.

**Table 2:** A summary of our findings regarding malaria medicine and vaccine R&D.

	Early Stage Discovery	Late Stage Discovery	Clinical Trials
Who pays?	Non-industry (99.8% of investments)	Non-industry (64–80% of investments)	Non-industry (78–89% of clinical trials)
Who performs?	Non-industry (88% of sample articles)	Non-industry (87% of sample articles)	Non-industry (85–88% of clinical trials)
Why do they perform?	Contributing to malaria eradication (72% of survey respondents), enjoyment (67%) and networking with other malaria researchers (52%)	Enjoyment (79% of survey respondents) and contributing to malaria eradication (71%)*	(Included in late stage discovery)
Is there collaboration?	Yes (83% of sample articles)	Yes (99% of sample articles)	(Included in late stage discovery)
Are results open access?	No (28% of sample articles)	Yes (65% of sample articles)	(Included in late stage discovery)
Are results in the public domain (i.e. not patented)?	No (33% of survey respondents)	Potentially* (64% of survey respondents)	(Included in late stage discovery)
Who owns patents?	(Typically too early for patents)	Both, with non-industry owning 54–56% of patents	(Included in late stage discovery)
Are physical results available for external researchers?	No (36% of survey respondents)	Potentially not* (36% of survey respondents)	(Included in late stage discovery)

* Small sample size

### Early Stage Malaria Drug Discovery

The G-Finder database reports that 99.8% of all basic research funding directed towards malaria is from non-industry organizations, with a total of US$ 557 million between 2007 and 2011.

An analysis of early stage research articles confirms that the majority (85% or n = 137) of articles have been financed from non-industry organizations, although seven of these articles acknowledged industry donations of materials. 10% (n = 16) did not state their funding source and did not reply when requested via e-mail to identify their funding source. 2.5% (n = 4) stated their funding source as industry. The remaining 2.5% (n = 4) stated their funding source as both industry and non-industry.

The articles’ corresponding authors were overwhelmingly affiliated with non-industry organizations (94% or n = 152). This did not differ much when all authors’ affiliations were taken into account; the majority (88% or n = 143) of articles were written by author groups only affiliated with non-industry organizations. The majority (83% or n = 134) of articles were written by researchers with different affiliations, 71% (n = 115) having three or more affiliations. Although only 37% (n = 59) of the authors represented cross-continental collaboration. 28% (n = 45) of the articles were available open access.

38% (n = 54) of the corresponding authors responded to a short survey. (Our sample included only four corresponding authors employed by industry. No industry-affiliated authors replied to the survey.) The primary personal motivations for performing malaria research were: contributing to malaria eradication (72%), enjoyment (67%) and networking with other malaria researchers (52%). The primary anticipated employer’s motivations were: publicly-funded institution with a mandate to advance knowledge creation (57%), field of research is left to researcher’s discretion (51%) and social responsibility (37%).

44% of respondents stated that their malaria research is patented or will be patented. A further 19% stated that the decision to patent has not yet been determined. The majority (73%) of the patents is applied for at the time of finding and optimizing lead compounds. 29% stated that they do not plan to patent their research, and 4% stated that their research is not patentable. Therefore, 33% of this research is in the public domain. (Although one could argue that the 19% that are considering patenting but have not yet done so and have published at least part of their results, have in fact placed their results in the public domain.)

A majority (75%) of the research projects collected biological samples or physically generated new chemical or molecular compounds. Only 36% of these specimens/compounds have been made available to external researchers.

### Late Stage Malaria Drug and Vaccine Discovery and Clinical Trials

The G-Finder database does not differentiate between late stage discovery and clinical trials. These categories are lumped together by product type. G-Finder reports that 64% of all malaria *drug* development funding is from non-industry organizations, with a total of US$ 515 million between 2007 and 2011, and 80% of all malaria *vaccine* development funding is from non-industry organizations, with a total of US$ 436 million during the same period.

An analysis of articles reporting on clinical trials confirms that the majority of articles have been financed from non-industry organizations with 73% (n = 55) of the identified articles stating that their funding source was from a non-industry organization, although seven of these articles acknowledged industry donations of materials. 9% (n = 7) did not state their funding source and did not reply when requested via e-mail to identify their funding source. 9% (n = 7) stated their funding source as industry. The remaining 8% (n = 6) stated their funding source as both industry and non-industry.

The articles’ corresponding authors were overwhelmingly affiliated with non-industry organizations (96% or n = 72). This did not differ much when all authors’ affiliations were taken into account; the majority (87% or n = 65) of articles were written by author groups only affiliated with non-industry organizations. 99% of the articles (n = 74) of articles were written by researchers with different affiliations, 91% (n = 68) having three or more affiliations. 75% (n = 56) of the authors represented cross-continental collaboration. 65% (n = 49) of the articles were available open access.

20% (n = 14) of the corresponding authors of these articles responded to a short survey. (Our sample included only two corresponding authors employed by industry. No industry-affiliated authors replied to the survey.) The following survey results should be viewed only with interest, as the sample size is small. The primary personal motivations for performing malaria research were: enjoyment (79%) and contributing to malaria eradication (71%). The primary anticipated employer’s motivations were: publicly-funded institution with a mandate to advance knowledge creation (71%) and social responsibility (43%).

21% of respondents stated that their malaria research is patented or will be patented. A further 7% stated that the decision to patent has not yet been determined. The majority (75%) of the patents are applied for at the time of clinical trials. 57% stated that they do not plan to patent their research, and 7% stated that their research is not patentable. Therefore, 64% of this research is in the public domain, with the same caveat as previously that the 7% could arguably be included in this figure.

A majority (79%) of the research projects collected biological samples or physically generated new chemical or molecular compounds. Only 36% of these specimens/compounds have been made available to external researchers.

91% (n = 178) of the malaria-related clinical trials in the WHO’s International Clinical Trials Registry Platform (ICTRP) were sponsored by non-industry sources and 92% (n = 180) were funded from non-industry sources. (The sponsor is defined as “the individual, organization, group or other legal entity which takes responsibility for initiating, managing and/or financing a study… The Primary Sponsor may or may not be the main funder.”) Of these trials, 78% (n = 152) were trials of drugs (n = 111) or vaccines (n = 41), and 88% (n = 134) were sponsored by non-industry organizations and 89% (n = 136) were funded by non-industry sources. When differentiated between drugs and vaccines these numbers are largely the same, i.e., 91% of the vaccine clinical trials were publicly funded and 89% of the drug clinical trials. 44% (n = 86) of the clinical trials had one or more collaborating organization (identified as secondary sponsor). Of the 15 industry-funded trials with a focus on drugs or vaccines, eight had secondary sponsors. All (but one) were public sector institutions. We compared the remaining seven trials against MMV’s Global Malaria Portfolio [21]. Five were included in MMV’s portfolio. This leaves only four trials without public sector support. However, one is seeking public funds [22] and another appears to have an academic partner [23]. The two remaining trials are both being sponsored by Indian private companies.

89% (n = 114) of the malaria-related clinical trials in the U.S. National Institutes of Health trial database (clinicaltrials.gov) were sponsored by non-industry sources. Of these trials, 77% (n = 98) were trials of drugs or vaccines, and 85% were sponsored by non-industry sources. 71% (n = 91) of the clinical trials had one or more collaborating organization. The website has filter capability based upon funding source (although this seems to actually mean if either the sponsor or the collaborator is in the private sector). 22% (n = 22) of the drug or vaccine trials were funded by industry or a combination of industry and non-industry.

There were 149 “malaria” patents secured through the US Patent and Trademark Office (http://patft.uspto.gov/) between 1993 and 2013. 56% (n = 84) of these patents are owned by non-industry actors. An additional seven patents are owned by a combination of private and public sector actors. The majority (n = 126) of the patents belong to four patent classes (424, 435, 514 and 530) which are all associated with drugs or vaccines. In these drug and vaccine-related patent classes 56% (n = 70) are owned by non-industry actors or non-individuals (with an additional four patents owned by a combination of private and public sector actors). These results are largely the same if broken down by drugs (56% owned by non-industry actors) and vaccines (54% owned by non-industry actors). A small percentage (17% or n = 26) of the total patents were owned by more than one organization.

Patent applications for malaria tell a similar story. There were 42 “malaria” patents applications filed through the US Patent and Trademark Office between 2011 and 2013. 60% (n = 25) of these applications were filed by non-industry actors. An additional five patents were filed by a combination of private and public sector actors. A minority (36% or n = 15) of the total patents were filed by more than one organization.

## Discussion

“*There are two main ways to finance supply of the public good new knowledge*. *The first is by government expenditure*. *The second is by the patent system*. *In the former case*, *taxpayers finance supply*. *In the latter case*, *consumers pay for it*. *Medical R&D is typically financed by a combination of the two approaches*, *with the balance between them being a social choice*.”[24]

This quote by Barret aptly describes the general perception of pharmaceutical innovation. However, in the case of malaria, both systems (government expenditure and patents) are being used concurrently by the not-for-profit sector. As we have demonstrated, public and philanthropic sectors are financing and performing malaria drug/vaccine discovery and development, and then restricting access through patents, ‘closed’ publications requiring journal fees and hidden away physical specimens, and, then finally also purchasing the end product, the antimalarials and related technologies. The Affordable Medicines Facility for malaria (AMFm), financed by UNITAID and The Global Fund to Fight AIDS, Tuberculosis and Malaria, disbursed co-payments for medicines distributed through the private sector of more than US$ 140 million in 2012 [25]. The U.S.’ President’s Malaria Initiative procured about 4.5 million malaria treatments for pregnant women and 72 million ACT treatments in 2012 [26]. The not-for-profit sector needs to attempt to get better value for its money. One way may be through a more open source business model given that most of the value chain is publicly and philanthropically funded. We outline such a model.

### Value Proposition

The value for applying a more “open source” approach to drug and vaccine discovery and development is to make the entire value chain more efficient through greater transparency which should lead to a reduction in duplicative research and may lead to further collaboration.

Increased transparency will eventually come about with the recently agreed formation of a Global Observatory on Health R&D to be hosted by WHO [27]. However, this will likely take some time to get up and running. In the meantime, MMV would be an ideal candidate to host malaria-related R&D information, including vaccine-related projects, since it is useful to have a single malaria database for ease-of-use. This would include providing:

- A registry of current malaria R&D projects so that researchers and donors can have a better understanding of ongoing research. This would include a summary of the research project, a contact individual and e-mail address, a timeline, the financing agency, a link to the project website, a categorization of where within the pharmaceutical value chain the research resides, and a number of keywords to assist in locating the project when searching.
- A registry of malaria R&D dry wells (i.e., previous research projects that did not lead to new technologies against malaria) so that researchers and donors know about past failures. This would include the same information as listed above in addition to a summary of the results.
- A biobank of active compounds/specimens so that researchers can collaborate with the same ingredients. MMV is already doing this by hosting the open access malaria box. MMV will send 400 diverse compounds identified to be active against malaria to any requestor who agrees to make their results openly available. The success of the malaria box has led to plans to develop a pathogen box [28].
- A regularly updated list of the current malaria-related R&D needs, as identified by WHO, MMV and others. This would assist R&D funders in prioritizing R&D funding requests as well as assist researchers in identifying potential research areas.

To make these registries a success, public and philanthropic financing organizations could support the capture of this data by stipulating to grantees that they must:

- Register their research projects before they can receive their funding.
- Analyze the dry wells and ongoing projects to ensure that their projects are not duplicative and identify areas for potential collaboration.
- Offer any relevant physical compounds or specimens to MMV for potential inclusion in their biobanks.
- Publish their articles open access.

MMV would, of course, require additional financing to take on this data management role.

Publishing open access is more costly for the individual researcher than traditional publishing since the author must pay a publication fee (for example, USD 1,350 to publish in PLOS ONE) [29]. This is a small price given the benefit that all readers can access and read the article free-of-charge. Potential savings could be achieved by restricting the use of public or philanthropic funds on the costs of patenting, which costs hundreds of thousands of US dollars if the patent is registered in multiple countries [30]. This does not mean that the institution cannot patent their findings. According to the U.S.’ Bayh-Dole Act (and its equivalents in other countries) institutions (and/or individuals) will still have the ability to patent their results. However, financing organizations can force them to use their own internal funds, thereby facilitating a pragmatic discussion about the likely cost/benefit of patenting products associated with market failure.

How does greater transparency lead to increased collaboration? We know that much of the research performed today is inaccessible until it is (hopefully) published. This lag means that researchers must rely on their own networks and informal communications to locate potential research collaborations. The respondents to our survey indicated that they want to collaborate with other researchers. When asked about the helpfulness of potential registries of previous and ongoing malaria research where projects are registered at the time of funding, they indicated that they were interested in using these registries to identify:

- Related research projects (79% of discovery researchers and 85% of clinical trial researchers);
- Potential collaborators (69% of discovery researchers and 85% of clinical trial researchers); and
- External research where the researcher may collaborate (62% of discovery researchers and 46% of clinical trial researchers).

### Market Segment/Revenue Model

The market for drugs or vaccines against malaria can be segmented by income brackets by country. Table 3 gives the number of suspected cases (in 2011) by type of malaria and by country classification.

**Table 3:** Suspected cases (in 2011) by type of malaria and income classification.

World Bank Country Income Classification	Suspected cases P. *falciparum* (2011)	Suspected cases P. *vivax* (2011)
Low income	83,085,131	4,249,798
Lower middle income	85,148,996	63,998,909
Upper middle income	10,019,627	12,114,649
High income	41,884	10,625
Total	178,295,638	80,373,981

WHO recommends that ACTs be used for the treatment of uncomplicated P. *falciparum* malaria [31]. Whereas chloroquine is still effective in most cases against P. *vivax* and ACTs are used as second-line treatment [31] except where chloroquine resistance has emerged, primarily in Indonesia, Malaysia, Myanmar and Viet Nam [32] which represents about 3.5 million cases. Therefore, the total annual market for ACTs can be roughly calculated at a little less than 200 million treatments. The Global Fund to Fight AIDS, Tuberculosis and Malaria has negotiated maximum prices between US$ 0.37 to 1.98 per course of ACT treatments, spanning across different medicines and treatment regimens [33]. A crude assumption could be a maximum payment for a new antimalarial of US$ 1.00 per course of treatment, therefore, an annual market of less than US$ 200 million. Of course the entire market will not be captured by one ACT, reducing potential revenues substantially. The cost of Phase III clinical trials varies widely from tens to hundreds of million dollars, depending upon the scale and complexity of the study [17]. Since return on investment is calculated on net profit (not revenues), it is quickly apparent that the expected return on a new antimalarial will, if any at all, be less than the interest earned on bank deposits, confirming that there are indeed market failures for malaria medicines.

Vaccines, however, are another case. A vaccine demonstrated to effectively prevent malaria (either P. *falciparum* or *vivax*) would be lucrative not only in low and middle income countries but also in high income ones due to tourist and military usage as well as expanding endemic areas due to mosquito migration as a consequence of global warming. The difficulty is developing a vaccine considered so efficacious that it is valuable to high income countries and can be a substitute for pharmacological prophylaxis. Since this cannot be determined until Phase III clinical trials are complete, the private sector still has an incentive to invest in malaria vaccine R&D with the knowledge that a partially effective vaccine can still be valuable to malaria endemic countries. Boston Consulting Group in 2005 estimated that the public market for a 50% efficacious vaccine against P. *falciparum* malaria (like RTS,S) in 2025 will be approximately 70 million people and for an 80% efficacious vaccine as high as 154 million, assuming no funding constraints [34]. If one assumed that a similar price could be achieved for a P. *falciparum* malaria as GAVI has negotiated for pneumococcal vaccines, i.e., US$ 3.50 per unit, then annual sales revenues for an RTS,S-like vaccine would be about US$ 250 million. (GSK has not yet proposed a price but has stated that it will sell at cost plus 5%.)

RTS,S has been co-developed through investments from GSK (reported investment of US$ 350 million with an additional US$ 260 expected [35]), the U.S.’ Walter Reed Army Institute of Research (which received US$ 3.8 million in 2012 for malaria vaccine research [36], so if the work began in 1982 this would roughly amount to over US$ 50 million cumulatively) and the Malaria Vaccine Initiative (reported investment of US$ 200 million from the Bill & Melinda Gates Foundation [35]). This investment by GSK demonstrates a belief that the market for a malaria vaccine could be a profitable one.

Our rough calculations demonstrate that the potential revenues attainable for a new antimalarial or a partially-effective vaccine will never be considered lucrative given the amount of R&D investment required. Therefore, the revenue model for an open source approach is not based upon earnings, but savings to ensure that the public/philanthropic sector is using its funds in the most effective manner.

### Cost Structure and Profit Potential

We will not go into great detail regarding the cost structure and profit potential, as these remain the same as today’s model. That is, the cost of discovering and developing new drugs is substantial, ranging from USD 200 million and up over USD 1 billion [37,38]. However, the cost of producing medicines, once they are approved for sale, is modest. A more open source approach will not change the overall economics here. The benefit of the model is to reduce the overall costs of discovery and development.

### Value Network

A formalized, publicly and philanthropically funded architecture for malaria R&D already exists in each position of the value network. Academia and non-profit research institutes largely perform the early stage research and then pass their results onto PDPs, including MMV and The PATH Malaria Vaccine Initiative, for further development through their portfolio approach. The European and Developing Countries Clinical Trials Partnership (EDCTP) finances clinical trials, with malaria as one of its priority diseases. UNITAID is mandated to support the commercialization of new antimalarials and related technologies. Lastly, the Global Fund to Fight AIDS, Tuberculosis and Malaria and the U.S.’ President’s Malaria Initiative support countries with their purchases of antimalarials and related technologies. Only HIV and tuberculosis share this comprehensive, publicly and philanthropically funded architecture to facilitate the development and launch of new medicines and vaccines. The more efficient the overall network can operate, the more value that will be created through public health impact.

### Competitive Strategy

Competitive strategy focuses on how innovators gain and hold advantage over rivals. In the case of drugs and vaccines for malaria, it concerns how researchers and developers protect their R&D results and technologies from being appropriated by competitors. Three principle mechanisms used here are patents, exclusivities and WHO prequalification. We will cover these in reverse order.

WHO prequalification started off as a quality assurance mechanism for UN agencies (like UNICEF) to ensure that they were purchasing high quality products. However, other donors and agencies (like the Global Fund to Fight AIDS, Tuberculosis and Malaria) have followed the UN’s example and also require WHO prequalification for bulk purchasing. There are no restrictions on the number of manufacturers that can be prequalified per product. However, the desirability to invest in clinical trials and prequalification diminishes with the number of manufacturers already prequalified for the particular product. Only one malaria medicine (with the same formulation and strength) has attracted four prequalified manufacturers. The next highest is two prequalified manufacturers per product/formulation/strength. Since the main purchasers require WHO prequalification, it is a significant competitive advantage to be one of the first ACT manufacturers to receive prequalified status.

Market and data exclusivities are awarded automatically upon regulatory approval from specific regulatory agencies, including FDA, EMA and the Japanese regulatory authority (those agencies where law mandates the award of exclusivities). Data exclusivity restricts other organizations from seeking regulatory approval of a product using the same clinical trial data as the originating organization for a specified period of time (from 5–8 years for new chemical entities and up to 12 years for biological products). Market exclusivity may be granted for products that meet certain classifications (new pediatric medicines or orphan medicines). There is no exclusivity granted through WHO prequalification. Exclusivities are largely applicable to potential malaria vaccines, as these would go through regulatory approval in Europe, Japan or the U.S.

The standard protection measure for pharmaceutical R&D is the patent, used by both the public sector (including academia, national research institutes and PDPs) and the private sector. MMV is listed in The World Intellectual Property Organization’s patent database, Patentscope, as owning 15 patents, which encompass four different patent groups. Three of these are filed through the global Patent Cooperation Treaty, which includes the African Regional Intellectual Property Organization (ARIPO). This initial filing must be followed up by national or regional filings in order to obtain the actual patent for the designated country. Since ARIPO does not have up-to-date data in Patentscope, it is not possible to determine whether MMV has filed in African countries, and MMV does not itemize its patent ownership or costs in its annual reports.

Based upon our results, we wonder if patenting of malaria treatments by non-industry organizations, like MMV, is justified. The cost of patenting is not immaterial, requiring patent applications to be filed and followed up in each individual country. Additionally the purpose of a patent is to protect against infringement, through costly lawsuits. The question is—why would a publicly or philanthropically funded body that has developed a new medicine to treat malaria (where there is market failure) want to monopolize a medicine with no or very modest revenues?

One possibility is that they may want to maintain control of the new medicine to ensure that only quality-assured manufacturers are allowed to produce it. However, this should rather be controlled through the regulatory approval process. Another argument is that a patent is necessary to incentivize the participation of industry in commercialization. Industry expertise is indeed valuable in development and necessary for manufacturing and distribution. However, early WHO prequalification may also be a large enough incentive to gain the participation of industry, particularly generic manufacturers or those located in low and middle-income countries. Many of the manufacturers that MMV works with are from middle-income countries. For a Chinese or Indian manufacturer a modest profit may be sufficient if they receive financial (and potentially technical) assistance for WHO prequalification. This too can be achieved without patents. MMV can partner with UNITAID and tender out WHO prequalification support to the most qualified applicant.

This is not to say that pharmaceutical manufacturers that develop a new antimalarial should not be allowed to patent. Of course, they should protect their private investment where relevant. But the utility of patenting publicly-funded innovation is questionable when the main aim of these public funds is to improve global public health.

### Business model

To sum up, our analysis finds that malaria R&D could benefit from much greater transparency and openness. Since malaria R&D is largely publicly and philanthropically funded and the end result purchased by these same governments or philanthropies, efficiencies gained mean that these monies can be used elsewhere, potentially funding additional R&D or purchasing more medicines or vaccines. We would encourage publicly-funded institutions to re-evaluate their financing of patents and examine if similar benefits can be achieved with first-mover status on WHO prequalification.

## Conclusions

We have evaluated existing efforts to discover and develop new drugs and vaccines for malaria to determine whether malaria R&D would benefit from an open source approach and how such a business model may operate. Our results demonstrate that mainly the public and philanthropic sectors are financing and performing malaria drug/vaccine discovery and development, but then restricting access to the research outputs through patents, journal fees and hidden away physical specimens. This makes little sense since it is the same public and philanthropic sector that purchases the drugs and vaccines. We recommend that a more “open source” approach is taken by making the entire value chain more efficient through greater transparency which may then lead to further collaboration.

We commend the main malaria medicines product development partnership, MMV, for its proactive stance on the use of open source approaches [18,28]. It openly shares hundreds of compounds identified to be active against malaria and is also exploring an open source research project with Open Source Malaria, a project aiming to improve some specific molecules that are now in the public domain [39]. Our analysis is in no way a critique of MMV, an organization that has effectively revived malaria R&D to unprecedented levels. We rather hope that our findings will give MMV and other R&D organizations as well as malaria R&D funders, new ideas regarding how to get more value for their money thereby creating more impact for future patients.

## Supporting Information

S1 Appendix Surveys (DOCX)
