# Supplementary material for: An Open Source Business Model for Malaria
Source: PLoS One. 2015 Feb 6;10(2):e0117150. doi: 10.1371/journal.pone.0117150 (PMC4320066; doi:10.1371/journal.pone.0117150)
Supplement: S1 Appendix — (DOCX) [file pone.0117150.s001.docx]

**Appendix S1: Surveys**

**Survey for researchers who have published on drug discovery for malaria**

1. What is your personal motivation for performing malaria research? (Please check all that apply.)

- I enjoy performing malaria research.
- I am formally educated to perform malaria research.
- I believe that performing malaria research will help me to get my first job.
- I believe that performing malaria research will assist the progression of my career.
- I am interested in learning more about malaria and therefore perform malaria research to learn more about the topic.
- I am interested in being a part of a network of malaria researchers, for example to be able to participate actively at related conferences with my peers.
- My family and friends are at risk from malaria. I want to make a difference in their lives.
- I want to improve the world by doing my part to eventually eradicate malaria.
- I did not select malaria as my field of research – my employer has chosen malaria research as a part of my duties.
- I perform malaria research because that was the funding that I was able to secure.
- Other (free text field)
- I do not perform malaria research. (Go to end.)

1. What type of organization is your employer? (Please select the one that best applies.)

- A university or college
- A government research institute
- A for-profit company (for example, a pharmaceutical company)
- A private non-profit research institute (for example, foundation-based)
- I am self-employed. (Go to Question 4.)
- I am unemployed. (Go to Question 4.)
- I am retired. (Go to Question 4.)
- I am a student. (Go to Question 4.)
- Other (free text field)

1. What do you believe is your employer’s motivation for performing malaria research? (Please check all that apply.)

- My employer is a publicly-funded institution with a mandate to advance knowledge creation.
- My employer believes that there is a potential profit in researching malaria.
- My employer believes that it has a social responsibility to research malaria in order to improve health in low-income countries.
- There is external funding readily available to perform malaria research.
- My employer educates students regarding malaria and therefore my research assists in the students’ formal education.
- My employer is located in a malaria-endemic country, and it is a national priority to research malaria.
- My employer leaves my field of research up to my own discretion.
- Other
- My malaria research activities are not a part of my paid job.

1. Have you or your organization applied for any patents on your malaria research, or plan to apply for patents on your malaria research?

- Yes (Go to Question 5.)
- No (Go to Question 6.)
- I/we have not yet decided. (Go to Question 5.)
- I do not know. (Go to Question 6.)
- My research is not patentable. (Go to Question 6.)

1. What stage would you designate the research that you have patented or plan to patent? (Please select the one that best applies.)

- Basic research (i.e., undirected research into the mechanisms and organisms that cause malaria)
- Target identification and validation (i.e., identifying and validating points where drugs or vaccines can intervene to disrupt malaria, or where diagnostics could detect malaria)
- Finding and optimizing lead compounds to ensure that they possess the properties needed to make an effective drug or vaccine
- Developing processes for making candidate drugs, vaccines or diagnostics in large, affordable quantities
- Clinical trials (i.e., determining the efficacy and safety of a drug/vaccine in people or the efficacy of a diagnostic)
- Other
- None of the above

1. Did your research collect any biological samples (i.e., blood specimens, parasite specimens, etc.) or physically generate new chemical or molecular compounds?

- Yes (Go to Question 7.)
- No (Go to Question 8.)
- I do not know. (Go to Question 8.)

1. Have you made these specimens/compounds available to external researchers, for example, through a biobank or another repository?

- Yes
- No
- I do not know.

1. One area that we are considering is the helpfulness of registries of previous and ongoing malaria research. The idea is that research projects would be registered once they are funded or started. Therefore, in theory, you could be alerted to specific, newly initiated research projects in a topic of your choice.

What could be the benefits of this registry? (Please check all that apply.)

- The registry could help me to become aware of related research projects.
- The registry could help me to identify gaps within my field of research.
- The registry could help me to determine my future research projects.
- The registry could help me to identify potential collaborators for my research.
- The registry could identify external research that I might be interested in collaborating on.
- The registry could reduce duplication of research.
- Other (free text field)
- I do not see any benefits of such a registry.
- I do not know.

1. We are also examining the potential of placing preliminary research results on a publicly-available website for comment or review. Prominent journals have indicated that sharing research results in this fashion is acceptable and do not preclude the research from later publication.

Would you consider sharing your preliminary research results on a publicly-available website?

- Yes (Go to Question 10.)
- No (Go to Question 11.)
- I do not know. (Go to Question 11.)

1. What types of results might you be willing to share? (Please check all that apply.)

- Raw results data
- Summarized results data
- Working papers in development
- Other (free text field)
- I do not know.

1. Do you have any additional comments? (Free text)

**Survey for researchers who have published on drug development for malaria**

1. What is your personal motivation for performing malaria research and development? (Please check all that apply.)

- I enjoy performing malaria research.
- I am formally educated to perform malaria research.
- I believe that performing malaria research will help me to get my first job.
- I believe that performing malaria research will assist the progression of my career.
- I am interested in learning more about malaria and therefore perform malaria research to learn more about the topic.
- I am interested in being a part of a network of malaria researchers, for example to be able to participate actively at related conferences with my peers.
- My family and friends are at risk from malaria. I want to make a difference in their lives.
- I want to improve the world by doing my part to eventually eradicate malaria.
- I did not select malaria as my field of R&D – my employer has chosen malaria research as a part of my duties.
- I perform malaria research because that was the funding that I was able to secure.
- Other (free text field)
- I do not perform malaria research. (Go to end.)

1. What type of organization is your employer? (Please select the one that best applies.)

- A university or college
- A hospital or medical facility
- A government research institute
- A for-profit company (for example, a pharmaceutical company)
- A private non-profit research institute (for example, foundation-based)
- I am self-employed. (Go to Question 4.)
- I am unemployed. (Go to Question 4.)
- I am retired. (Go to Question 4.)
- I am a student. (Go to Question 4.)
- Other (free text field)

1. What do you believe is your employer’s motivation for performing malaria R&D? (Please check all that apply.)

- My employer is a publicly-funded institution with a mandate to advance knowledge creation.
- My employer believes that there is a potential profit in malaria R&D.
- My employer believes that it has a social responsibility to perform malaria R&D in order to improve health in low-income countries.
- There is external funding readily available to perform malaria R&D.
- My employer educates students regarding malaria and therefore my research assists in the students’ formal education.
- My employer is located in a malaria-endemic country, and it is a national priority to perform malaria R&D.
- My employer leaves my field of R&D up to my own discretion.
- Other
- My malaria R&D activities are not a part of my paid job.

1. Have you or your organization applied for any patents on your malaria R&D activities, or plan to apply for patents on your malaria R&D activities?

- Yes (Go to Question 5.)
- No (Go to Question 6.)
- I/we have not yet decided. (Go to Question 5.)
- I do not know. (Go to Question 6.)
- My research is not patentable. (Go to Question 6.)

1. At what stage did you or your employer patent your R&D or plan to patent your R&D? (Please select the one that best applies.)

- Basic research (i.e., undirected research into the mechanisms and organisms that cause malaria)
- Target identification and validation (i.e., identifying and validating points where drugs or vaccines can intervene to disrupt malaria, or where diagnostics could detect malaria)
- Finding and optimizing lead compounds to ensure that they possess the properties needed to make an effective drug or vaccine
- Developing processes for making candidate drugs, vaccines or diagnostics in large, affordable quantities
- Clinical trials (i.e., determining the efficacy and safety of a drug/vaccine in people or the efficacy of a diagnostic)
- Other
- None of the above

1. Did your R&D collect any biological samples (i.e., blood specimens, parasite specimens, etc.) or physically generate new chemical or molecular compounds?

- Yes (Go to Question 7.)
- No (Go to Question 8.)
- I do not know. (Go to Question 8.)

1. Have you made these specimens/compounds available to external researchers, for example, through a biobank or another repository?

- Yes
- No
- I do not know.

1. One area that we are considering is the helpfulness of registries of previous and ongoing malaria R&D. The idea is that research projects would be registered once they are funded or started. Therefore, in theory, you could be alerted to specific, newly initiated research projects in a topic of your choice.

- What could be the benefits of this registry? (Please check all that apply.)
- The registry could help me to become aware of related research projects.
- The registry could help me to identify gaps within my field of research.
- The registry could help me to determine my future research projects.
- The registry could help me to identify potential collaborators for my research.
- The registry could identify external research that I might be interested in collaborating on.
- The registry could reduce duplication of research.
- Other (free text field)
- I do not see any benefits of such a registry.
- I do not know.

1. We are also examining the potential of placing preliminary research results on a publicly-available website for comment or review. Prominent journals have indicated that sharing research results in this fashion is acceptable and do not preclude the research from later publication.

Would you consider sharing your preliminary research results on a publicly-available website?

- Yes (Go to Question 10.)
- No (Go to Question 11.)
- I do not know. (Go to Question 11.)

1. What types of results might you be willing to share? (Please check all that apply.)

- Raw results data
- Summarized results data
- Working papers in development
- Other (free text field)
- I do not know.

1. Do you have any additional comments? (Free text)
